# Microstructure and Mechanical Properties of the As-Cast and As-Homogenized Mg-Zn-Sn-Mn-Ca Alloy Fabricated by Semicontinuous Casting

**DOI:** 10.3390/ma11050703

**Published:** 2018-04-29

**Authors:** Xing Lu, Guoqun Zhao, Jixue Zhou, Cunsheng Zhang, Junquan Yu

**Affiliations:** 1 Key Laboratory for Liquid-Solid Structural Evolution and Processing of Materials (Ministry of Education), Shandong University, Jinan 250061, China; luxing@mail.sdu.edu.cn (X.L.); zhangcs@sdu.edu.cn (C.Z.); junquan.yu@imperial.ac.uk (J.Y.); 2 Shandong Key Laboratory for High Strength Lightweight Metallic Materials, New Materials Research Institute, Shandong Academy of Sciences, Jinan 250014, China; zhoujx@sdas.org

**Keywords:** magnesium alloy, semicontinuous casting, homogenization treatment, microstructure, mechanical properties

## Abstract

In this paper, a new type of low-cost Mg-3.36Zn-1.06Sn-0.33Mn-0.27Ca (wt %) alloy ingot with a diameter of 130 mm and a length of 4800 mm was fabricated by semicontinuous casting. The microstructure and mechanical properties at different areas of the ingot were investigated. The microstructure and mechanical properties of the alloy under different one-step and two-step homogenization conditions were studied. For the as-cast alloy, the average grain size and the second phase size decrease from the center to the surface of the ingot, while the area fraction of the second phase increases gradually. At one-half of the radius of the ingot, the alloy presents the optimum comprehensive mechanical properties along the axial direction, which is attributed to the combined effect of relatively small grain size, low second-phase fraction, and uniform microstructure. For the as-homogenized alloy, the optimum two-step homogenization process parameters were determined as 340 °C × 10 h + 520 °C × 16 h. After the optimum homogenization, the proper size and morphology of CaMgSn phase are conducive to improve the microstructure uniformity and the mechanical properties of the alloy. Besides, the yield strength of the alloy is reduced by 20.7% and the elongation is increased by 56.3%, which is more favorable for the subsequent hot deformation processing.

## 1. Introduction

As one of the lightweight metal structural materials, magnesium alloys are gaining more attention and applications in the fields of automobile, rail transportation, aerospace, defense, 3C, etc., due to their high specific strength, superior fuel efficiency, good machinability and damping ability [1,2,3]. The magnesium alloy, with low-cost and good comprehensive mechanical properties, has always been in urgent demand for industry. Addition of rare-earth (RE) alloying elements into magnesium alloys can improve the comprehensive mechanical properties of magnesium alloys. However, the rare and expensive RE alloying elements lead to the high cost of Mg-RE alloys. Moreover, the high relative atomic mass of RE elements reduces the lightweight advantages of magnesium alloys with high content RE elements [4]. Therefore, the development of RE-free magnesium alloys with good comprehensive mechanical properties is gradually becoming a significant research focus.

Mg-Zn-Sn based alloys, as one of the low-cost and heat-resistant RE-free magnesium alloys, have attracted much attention in recent years. Compared with RE elements or other valuable elements (such as Li, Ag, Zr), Zn and Sn elements have an obvious price advantage. Previous researches have shown that the Mg-Sn-Zn based alloys exhibit the preferable and possible combination of strength and ductility both at room temperature and elevated temperatures [5,6,7]. Therefore, the Mg-Zn-Sn-based alloys become potential heat-treatable alloys for future commercial applications [8,9] and are able to achieve a high strength after deformation [10]. Wei et al. [11] indicated that the addition of minor Ca element to the alloy can significantly refine grains and grain-boundary compounds, thus improving the alloy’s hot workability. Moreover, the Ca element and the Sn element facilitate the formation of a more stable phase CaMgSn than Mg_2_Sn, thus further improving the thermal stability and creep resistance of the alloy [12,13,14]. Besides, the addition of minor Mn element can remove Fe, and improve the corrosion resistance and creep resistance of magnesium alloys [15].

At present, the Mg-Zn-Sn alloys ingots are mainly produced by conventional steel and sand mold casting [16,17,18,19]. However, the conventional casting process has some disadvantages such as elements segregation and emergence of casting shrinkage cavities and porosity, leading to low strength or poor ductility. For example, the tensile strength, yield strength, and elongation of the as-cast Mg-2Sn-1Ca-2Zn alloy are only 57 MPa, 42 MPa and 0.9%, respectively [20]. The elongation of as-cast Mg-6Zn-3Sn-2Al alloy is 6.5% [21]. Semicontinuous casting process can overcome the above disadvantages to a certain extent, and can produce larger-scale ingots with lower scrap rate, higher productivity and more uniform microstructure, compared with conventional casting [22,23]. However, the microstructure and mechanical properties of Mg-Zn-Sn based alloys fabricated by semicontinuous casting have been rarely studied.

The homogenization treatment process plays a key role in the subsequent hot deformation of as-cast alloys. Reasonable homogenization treatment can dissolve undesirable second phases, eliminate micro-segregations, and improve microstructure uniformity [24]. Besides, homogenization treatment can increase the hot workability of the alloy and extend its deformable range [25]. The research results of Miao et al. [26] showed that the elongation and corrosion resistance of the as-extruded Mg-2.4Zn-0.8Gd alloy are improved after homogenization treatment. Peng et al. [27] concluded that, with the increase of homogenization time, the strength of Mg-8Li-3Al-Y alloy increases, but the elongation firstly increases and then decreases. Liu et al. [28] proposed that the 14H long periodic stacking ordered (LPSO) structure formed during isothermal homogenization can lead to refined microstructure and weak texture after hot extrusion. For alloys containing heat-resistant phase, the size and morphology of the phase have a significant influence on the mechanical properties of the alloy. CaMgSn phase is a heat-resistant phase and difficult to be dissolved. However, the size and morphology of the CaMgSn phase can be changed during homogenization. When this heat-resistant phase presents proper size and morphology, it is conducive to improve the microstructure and increase the mechanical properties of the alloy. Pan et al. [20] developed Mg-2Sn-1Ca and Mg-2Sn-1Ca-2Zn alloys with good strength and ductility by using conventional casting, homogenization and extrusion, and the results showed that fine CaMgSn phase contributes to increasing the strength of the extruded alloy. Yang et al. [29] also indicated that the refined CaMgSn phase improves the alloy’s comprehensive mechanical properties. However, few researches have been reported on optimization of homogenization treatment process of the alloy containing CaMgSn phase.

A new type of low-cost magnesium alloy was developed in this research, and the large-scale ingots with the diameter of 130 mm and the length of 4800 mm fabricated by semicontinuous casting were presented in this paper. The distribution characteristics and the uniformity of the microstructure (such as grain, second phase and texture) of the semicontinuous ingot at different areas were investigated. The effect of different parameters of one-step and two-step homogenization treatment on the dissolution of heat-resistant phase was studied and the relationship between the microstructure (such as grain size, heat-resistant phase morphology) and mechanical properties of the as-homogenized alloy was revealed. These results are helpful to recognize the distribution of the microstructure of the semicontinuous ingot and provide necessary information for determining the optimum parameters of homogenization treatment.

## 2. Experimental Procedures

### 2.1. Semicontinuous Casting

**Figure 1:**
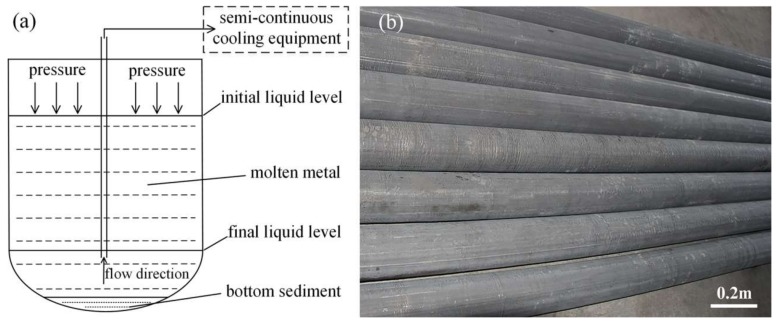
(**a**) Schematic illustration of the semicontinuous casting process; (**b**) the photo of ZTMX3100 alloy ingots fabricated by semicontinuous casting.

Pure magnesium (>99.99 wt %), pure Zn (>99.99 wt %), pure Sn (>99.99 wt %), Mg-10.15Mn (wt %) and Mg-20.23Ca (wt %) master alloys were melted at 720 °C and then kept for 1 h in the crucible under a mixed atmosphere (CO_2_:SF_6_ = 100:1). As shown in the schematic illustration of the casting process in Figure 1a, the molten metal was delivered from the bottom of the airtight crucible under pressure to the semicontinuous cooling equipment, and then directly cooled for directional solidification, thus preventing the mix of air and bottom sediment. The casting equipment worked with the casting speed of 110 mm/min during the semicontinuous direct-chill casting. The large ZTMX3100 alloy ingots with the diameter of 130 mm and the length of 4800 mm were fabricated, as shown in Figure 1b. The chemical compositions of the ingot at different areas were analyzed by inductively coupled plasma spectrometer (ICP-AES, Agilent Technology 725-ES, Agilent Technologies Ltd., Santa Clara, CA, USA), with average composition of Mg-3.36Zn-1.06Sn-0.33Mn-0.27Ca (wt %) (named as ZTMX3100). The average density of the alloy is 1.782 g/mm^3^, which is measured by the Archimedes principle method.

### 2.2. Homogenization Treatment Process

To determine the optimum homogenization process, one-step homogenization, and two-step homogenization at different temperatures and different times were conducted. The samples for homogenization treatment were taken along the axial direction from the 1/2 radius of the ingot. In one-step homogenization, the samples were heated to 460 °C, 500 °C, and 540 °C and kept for 4 h, 8 h, 16 h, and 24 h, respectively. Two-step homogenization includes the low-temperature stage and high-temperature stage. At low-temperature stage, the samples were heated to 340 °C and then kept for 2 h, 6 h, 10 h, and 12 h, respectively, while at high-temperature stage, the samples were heated to 520 °C and 560 °C, and kept for 4 h, 8 h, 16 h, and 24 h, respectively.

### 2.3. Microstructure and Mechanical Properties

**Figure 2:**
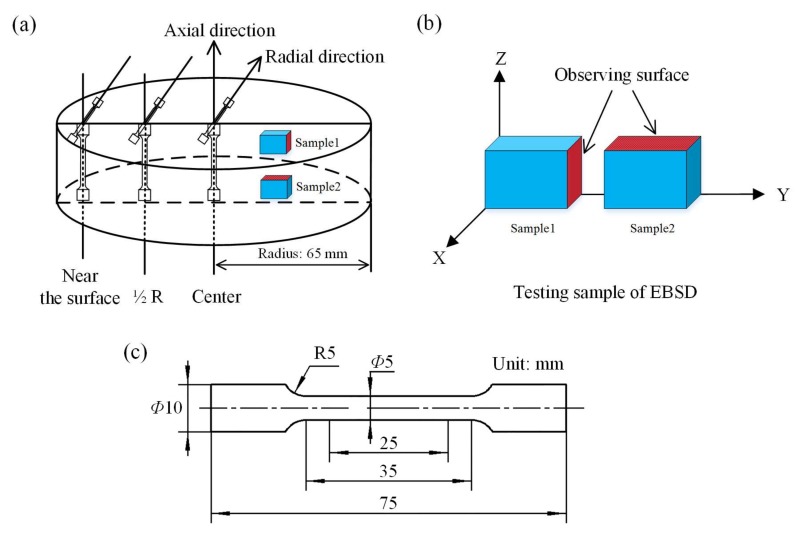
(**a**) Schematic illustration of the tensile samples and electron backscattered diffraction (EBSD) samples taken along axial and radial directions; (**b**) EBSD testing sample and its coordinate system; (**c**) the dimensions of the dog-bone shaped tensile sample.

The microstructure of the as-cast and as-homogenized alloys was observed in an optical microscope (OM, Olympus GX51, Olympus Ltd., Tokyo, Japan), and a field emission scanning electron microscopy (FE-SEM, HITACHI SU-70, Hitachi Ltd. Tokyo, Japan) equipped with an energy dispersive X-ray spectrometer (EDS, HORIBA EX-250, HORIBA Ltd., Kyoto, Japan). In SEM test, the beam intensity was set as 100 nA, and the voltage was set as 15 kV. The average grain size was measured by using Image-Pro Plus software (Version 6.0, Media Cybemetics Co., Rockville, MD, USA). At least 800 grains from different optical micrographs were analyzed for each condition. Samples for metallographic observation were taken from the center, 1/2 radius, and near the surface of the ingot, respectively. Samples for OM and SEM observations were etched with a mixed acid solution (1 g picric acid, 1 mL acetic acid, 20 mL ethanol and 2 mL distilled water). The samples for OM and SEM observations were taken along the axial direction of the ingot. The observation planes for OM and SEM were perpendicular to the axial direction. Microtextures of the as-cast alloy were conducted on an electron backscattered diffraction system (EBSD, Oxford Instruments-HKL, Oxford Instruments Ltd., Abingdon, UK) equipped with HKL Channel 5 analysis software (Version 5.09, Oxford Instruments Ltd., Abingdon, UK), with the field view of 1300 μm × 1300 μm and the step size of 4 μm. The modules “Tango” and “Mambo” of Channel 5 analysis software were used to analyze the obtained data. The ranges of low angular grain boundaries (LAGBs) and high angular grain boundaries (HAGBs) were 2–15° and 15–100°, respectively. The half-width was set as 10°, and the data clustering size was set as 5°. Figure 2a shows the schematic illustration of the EBSD samples taken from the 1/2 radius of the ingot. Figure 2b gives the EBSD testing sample and its coordinate system. Z direction is parallel to the axial direction. Phase identification and analysis were performed on an X-ray diffractometer (XRD, D/MAX-rC, Rigaku Ltd., Tokyo, Japan) with Cu-Kα radiation at voltage of 50 kV and current of 150 mA. The scanning range was between 20° and 90° and the scanning speed was set as 2°/min. The bulk sample for XRD was taken along the axial direction from the 1/2 radius of the ingot with diameter of 12 mm and height of 15 mm. Thermal stability was analyzed by using a differential scanning calorimetry (DSC, METTLER 1100LF, Mettler Toldeo Ltd., Greifensee, Zürich, Switzerland) at the heating rate of 20 K/min under a flowing argon atmosphere. The samples of the as-cast alloy for tensile tests were taken along axial and radial directions from the center, 1/2 radius, and near the surface of the ingot, respectively, while the samples of the as-homogenized alloy were taken along the axial direction from the 1/2 radius of the ingot. The schematic illustration of the tensile samples taken from different areas of the ingot is shown in Figure 2a. According to the testing standard of GB/T 16865-2013, the dimensions of the dog-bone shaped tensile sample are shown in Figure 2c, with original diameter of 5 mm and original gauge length of 25 mm. Tensile tests were conducted at room temperature by using a standard testing machine (CMT-5015, MTS Systems Co., Eden Prairie, MN, USA) with a strain rate of 1.0 × 10^−3^ s^−1^.

## 3. Results and Discussion

### 3.1. As-Cast Alloy

#### 3.1.1. Microstructure and Texture

**Figure 3:**
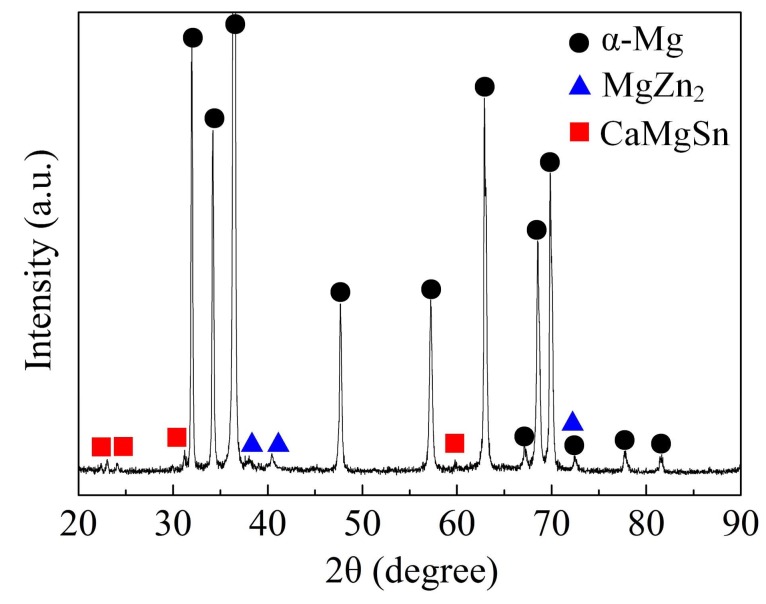
X-ray diffraction pattern of the as-cast ZTMX3100 alloy.

The examined chemical compositions of the ingot at different areas are shown in Table 1. It indicates that no obvious macro segregation of the solute elements occurs along radial direction, and the relatively homogeneous distribution of chemical composition is obtained. Figure 3 shows the XRD pattern of the as-cast ZTMX3100 alloy. It can be preliminarily concluded that there are at least three kinds of phases including α-Mg, MgZn_2_, and CaMgSn. This result is consistent with the investigations in other literature [30,31,32].

**Figure 4:**
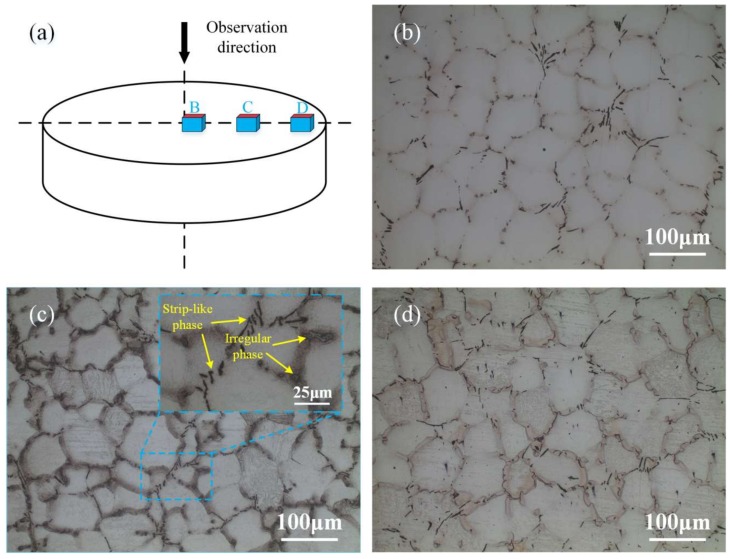
Optical micrograph of the as-cast ingot at different areas: (**a**) schematic diagram of the observed areas; (**b**) center; (**c**) 1/2 radius; and (**d**) near the surface of the ingot.

Figure 4a indicates the different areas of the ingot for microstructure observation. Figure 4b–d show the optical micrographs at the central, 1/2 radius, and near the surface of the ingot. It can be seen that the ingot is mostly composed of α-Mg matrix, long strip-like phase, and irregular phase mainly attaching along grain boundaries. From the center to the surface of the ingot, the average grain size and the second phase size decrease, while the area fraction of the second phase increases gradually. At the central, 1/2 radius, and near the surface of the ingot, the average grain sizes are 78.5 μm, 70.1 μm, and 65.2 μm, and their area fractions are 2.08%, 2.32%, and 2.85%, respectively. This phenomenon is reasonable, the faster cooling rate at surface of the ingot during semicontinuous casting impedes the primary crystal grain growth and hinders the formation of the second phase, thus leading to fine grain size and small second phase. At the same time, the faster cooling rate at surface of the ingot hinders atoms migrations and leads to a high degree of solute segregation, resulting in a higher area fraction of the second phases at the grain boundaries near the surface of the ingot [33]. No shrinkage cavity is found in the ingot.

**Figure 5:**
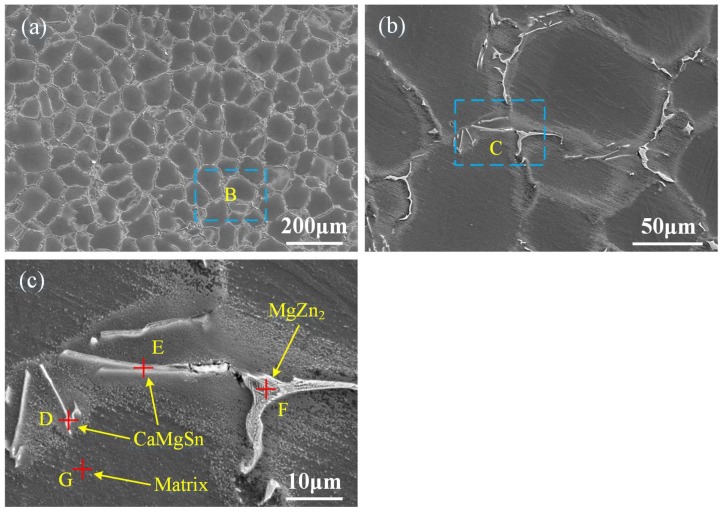
(**a**) Scanning electron microscopy (SEM) micrograph of the ingot; (**b**) the magnified image of the region marked B in (**a**); (**c**) the magnified image of the region marked C in (**b**).

Figure 5a shows the low-magnified SEM micrograph of the as-cast alloy. It can be seen that the α-Mg matrix is surrounded by the discontinuous long strip-like phase and irregular phase distributed homogeneously in the observed region. Figure 5b shows the magnified image of the region marked B in Figure 5a. It can be observed that dark long strip-like phase and white-bright irregular phase distribute along the grain boundaries. Figure 5c is the magnified image of the region marked C in Figure 5b. According to the results of EDS in Table 2, only large amount of the Mg element and small amount the Zn element can be detected at point G in the matrix. The atomic ratios of Ca/Sn at point D and point E are close to 1:1, demonstrating that the phase at point D and point E might be CaMgSn [7]. At point F, only the Mg element and the Zn element can be detected. In combination with XRD results, the phase containing Zn element is MgZn_2_, so it can be preliminarily assumed that the phase at point F might be MgZn_2_.

**Figure 6:**
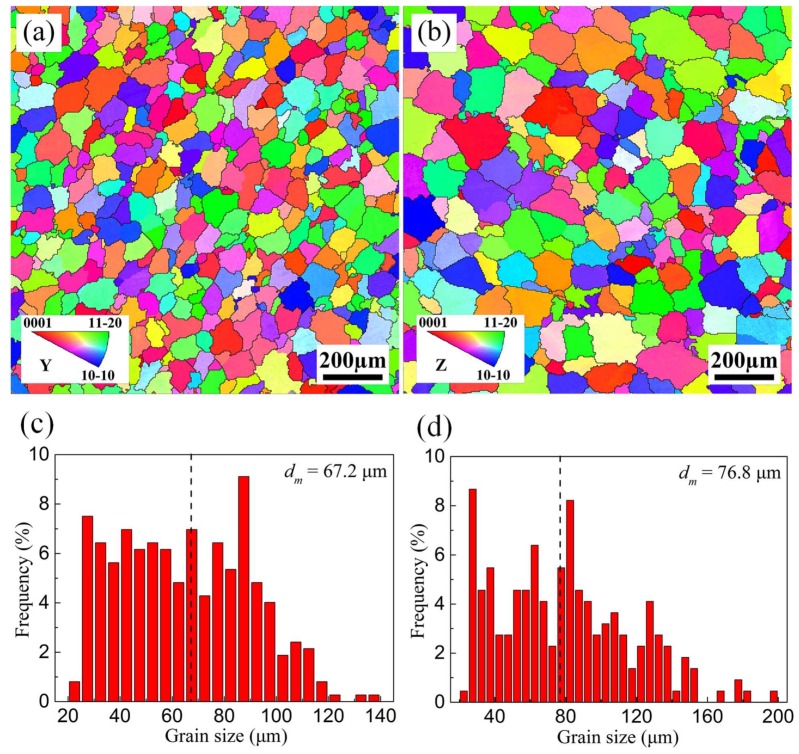
EBSD analyses results for the as-cast ZTMX3100 alloy: (**a**,**b**) inverse pole figure (IPF) maps with reference to Y and Z directions; (**c**,**d**) grain distribution histograms; (**a**,**c**) axial direction; (**b**,**d**) radial direction.

In order to check whether the semicontinuous alloy ingot exists a specific orientation or not, the electron back scattered diffraction (EBSD) tests were performed respectively along axial and radial directions at 1/2 radius of the ingot. In this paper, only high angle grain boundaries indicated by the black lines are displayed in Figure 6. The inverse pole figure (IPF) maps with reference to Y and Z directions are shown in Figure 6a,b, respectively. The grain distributes more uniformly and the grain size is finer along the axial direction than along the radial direction. As is shown in Figure 6c,d, the grain distribution histograms are obtained from EBSD data. Only the grains with high angular grain boundaries are counted by using the module “Tango” of Channel 5 analysis software. The dashed line in the grain distribution histogram presents the average grain size (*d*_m_). The grain along the axial direction mainly concentrates between 30 and 90 μm with the average size of 67.2 μm, while the grain along the radial direction mainly concentrates between 30 and 130 μm with the average size of 76.8 μm. The difference is because the grains along the axial direction have the same distance from the surface of the ingot with the same cooling condition and cooling rate, resulting in more uniform grain size and distribution. However, the grains along the radial direction have different distances from the surface of the ingot with various cooling conditions and cooling rates, thus leading to less uniform grain size and distribution.

**Figure 7:**
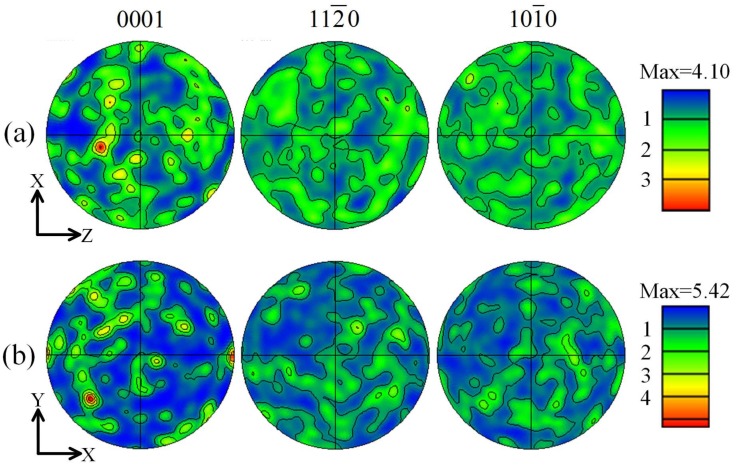
The (0001), (11−20) and (10−10) pole figures of the as-cast ZTMX3100 alloy at 1/2 radius of the ingot: (**a**) axial direction; (**b**) radial direction.

Figure 7a,b show the (0001), (11−20) and (10−10) pole figures of the as-cast alloy along axial and radial directions at 1/2 radius of the ingot. Figure 7 shows that the grain orientation of the as-cast alloy is random at different directions according to the texture analysis, which is in accordance with other investigations [33,34,35]. Besides, the maximum basal texture intensity along the axial direction is slightly smaller than that along the radial direction, valuing 4.10 and 5.42, respectively. This difference between the maximum basal texture intensities is attributed to different randomness of the grain orientations, which is dominated by the grain size and quantity within the observing area. The randomness of the grain orientations is statistically stronger with the finer and more grains in the observing area, thus leading to weaker texture intensity. As described in Figure 6, the grains along the axial direction are relatively finer and distribute more uniformly than that along the radial direction. Therefore, the maximum basal texture intensity along the axial direction is smaller than that along the radial direction. Borkar et al. [36] also revealed that the fine grains provide relatively more random orientations than the medium-to-large-sized grains, thus developing a weaker texture.

#### 3.1.2. Mechanical Properties and Fracture Behaviors

**Figure 8:**
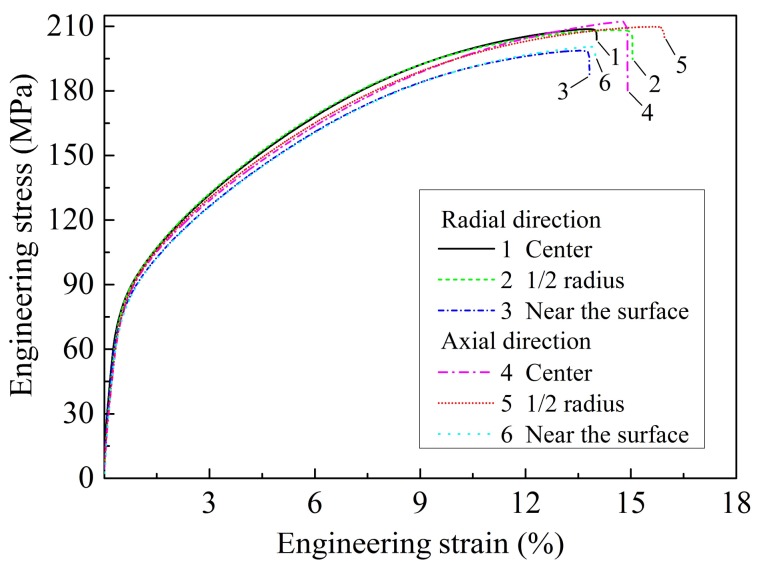
The engineering stress–strain curves of the as-cast ZTMX3100 alloy along radial and axial directions at different areas.

Figure 8 shows the tensile engineering stress–strain curves of the as-cast ZTMX3100 alloy along radial and axial directions at different areas. Table 3 gives the corresponding tensile properties of the tensile yield strength (YS), ultimate tensile strength (UTS), and elongation (EL), respectively. Compared with the radial direction, the mechanical properties of the alloy along the axial direction are slightly better. As can be seen in Figure 7, the pole figures of the as-cast alloy along axial and radial directions indicate that the grain orientation of the as-cast alloy is random. Therefore, the variation of the tensile properties is independent on texture. The difference in mechanical properties along radial and axial directions may be related to that the microstructure along the axial direction is more uniform than that along the radial direction, which is also consistent with the grain distribution shown in Figure 6c,d. Therefore, the samples along the axial direction present better mechanical properties.

In addition, the strength and ductility of the samples at the central and 1/2 radius of the ingot are all better than those near the surface of the ingot both along radial and axial directions. This experimental result can be explained that though the grain size near the surface of the ingot is relatively fine, the area fraction of the second phase on the grain boundary from the center to the surface of the ingot increases gradually, as is shown in Figure 4. These second phases lead to local stress concentration and are prone to fracture, eventually leading to lower strength and ductility of the samples near the surface of the ingot [37]. Moreover, the sample along the axial direction at 1/2 radius of the ingot presents the optimum comprehensive mechanical properties, with the YS, UTS, and EL of 84.5 MPa, 210.0 MPa, and 16.0%, respectively, which is attributed to the combined effect of relatively small grain size, low second phase fraction, and uniform microstructure.

**Figure 9:**
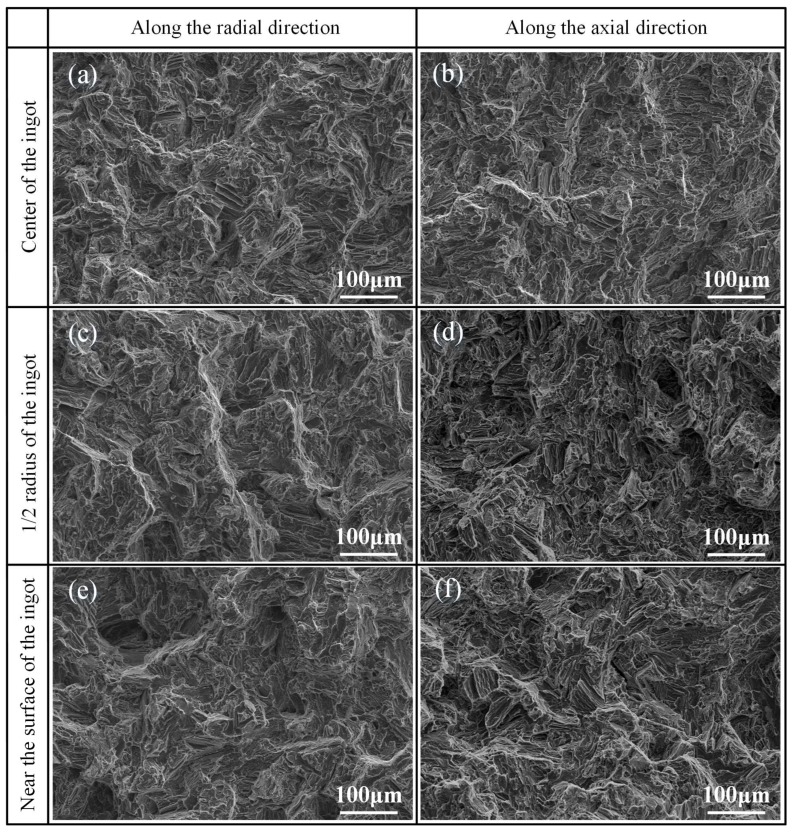
SEM micrographs of the fracture surface at different areas along radial and axial directions: (**a**,**b**) center of the ingot; (**c**,**d**) 1/2 radius of the ingot; (**e**,**f**) near the surface of the ingot; (**a**,**c**,**e**) along the radial direction; (**b**,**d**,**f**) along the axial direction.

Figure 9 shows the SEM micrographs of the fracture surface of the as-cast ZTMX3100 alloy along radial and axial directions at different areas. As can be seen in Figure 9a–f, the tearing edges and dimples are observed on the fracture surfaces both along radial and axial directions at different areas. Besides, the dimples along the axial direction at 1/2 radius of the ingot are found much larger and deeper than those at other areas, which is consistent with the tensile results that the sample tested at 1/2 radius of the ingot exhibits better ductility. This is because the relatively fine grains and small second phases can shorten the distance of dislocations slip and release the stress concentration at the grain boundaries [33], so that the sample along the axial direction at 1/2 radius of the ingot not only has a better elongation but also maintains the relatively high strength.

### 3.2. As-Homogenized Alloy

#### 3.2.1. Microstructure

**Figure 10:**
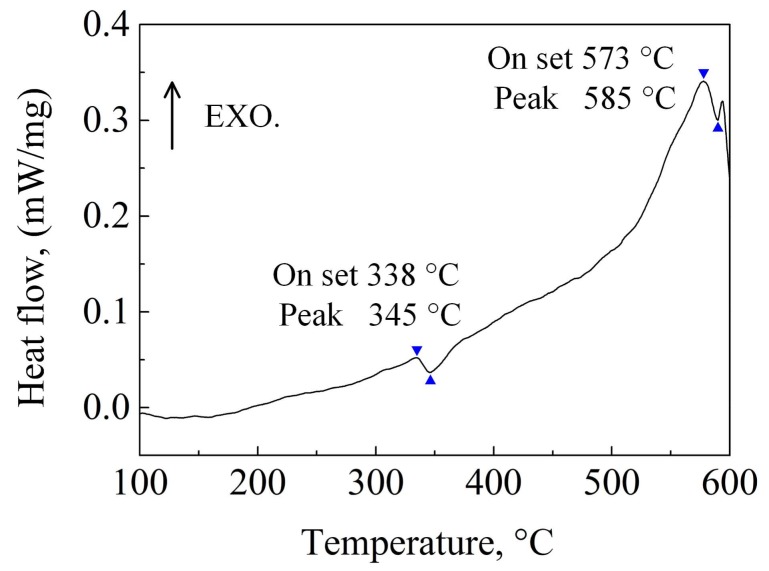
Differential scanning calorimetry (DSC) curve of the as-cast ZTMX3100 alloy.

Figure 10 shows the DSC curve of the as-cast ZTMX3100 alloy. It is found that the two endothermic peaks appear in the low temperature region and the high temperature region, corresponding to the dissolution of the low-melting-point phase MgZn_2_ and the heat-resistant phase CaMgSn, respectively. The peak temperatures approximately appear at 345 °C and 585 °C.

**Figure 11:**
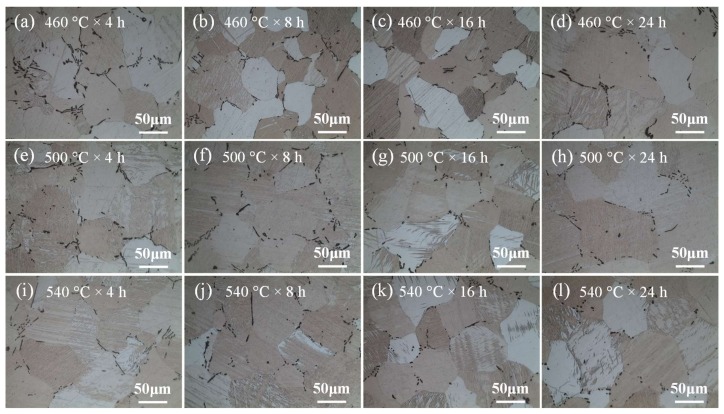
Optical microstructure of the samples for different homogenization temperatures and time in one-step homogenization.

**Figure 12:**
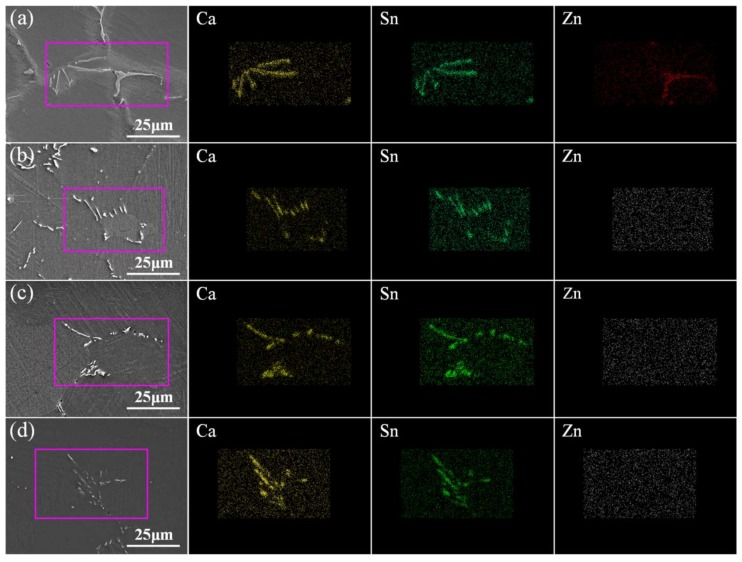
SEM micrographs and EDS element (Ca, Sn, Zn) mapping images of the as-cast and as-homogenized alloys: (**a**) as-cast; (**b**) 460 °C × 16 h; (**c**) 500 °C × 16 h; (**d**) 540 °C × 16 h.

In one-step homogenization, the samples were heated to 460 °C, 500 °C, and 540 °C and kept for 4 h, 8 h, 16 h, and 24 h, respectively. Figure 11 shows the optical microstructure of the samples in one-step homogenization. It can be seen from Figure 11a–d that the morphology of CaMgSn has no obvious change when homogenized at 460 °C. Besides, with the increase of homogenization time from 4 h to 24 h, the grain size changes little due to the pinning effect of long strip-like phase CaMgSn. The average grain sizes of Figure 11a–d are about 70–72 μm. As shown in Figure 11e–g,i–k, with the increase of homogenization temperature from 500 °C to 540 °C and the increase of homogenization time from 4 h to 16 h, the heat-resistant phase CaMgSn is dissolved from long strip-like into short rod-like or chain-like without obvious grain growth. The average grain sizes of Figure 11e–g are about 70.5 μm, 71.3 μm and 72.8 μm, and the average grain sizes of Figure 11i–k are about 71.9 μm, 72.3 μm and 73.9 μm, respectively. When the homogenization time exceeds 16 h, the grains grow up more obviously, with average grain sizes of about 75.6 μm and 79.9 μm, respectively, as shown in Figure 11h,l. Figure 12 gives the SEM micrographs and EDS element (Ca, Sn, Zn) mapping images of the as-cast and as-homogenized alloys. As indicated in Figure 12a, the Ca, Sn and Zn elements can be detected in the as-cast alloy. Besides, the distribution of the Zn element coincides with the position of the irregular phase MgZn_2_, while the distribution of the Sn and Ca elements coincides with the position of the long strip-like phase CaMgSn, thus further confirming the existence of MgZn_2_ and CaMgSn. However, as indicated in Figure 12b–d, MgZn_2_ phase is dissolved into the matrix after homogenization, the Zn element is not detected and only the Ca and Sn elements are detected in the as-homogenized alloys.

In order to further dissolve the heat-resistant phase CaMgSn and prevent over-burning of the low-melting-point phase, two-step homogenization with low-temperature stage and high-temperature stage is taken to study the microstructure evolution of the alloy. According to the DSC curve of the as-cast ZTMX3100 shown in Figure 10, the low-temperature stage homogenization is selected as 340 °C and the high-temperature stage homogenization is preliminarily selected as 560 °C.

**Figure 13:**
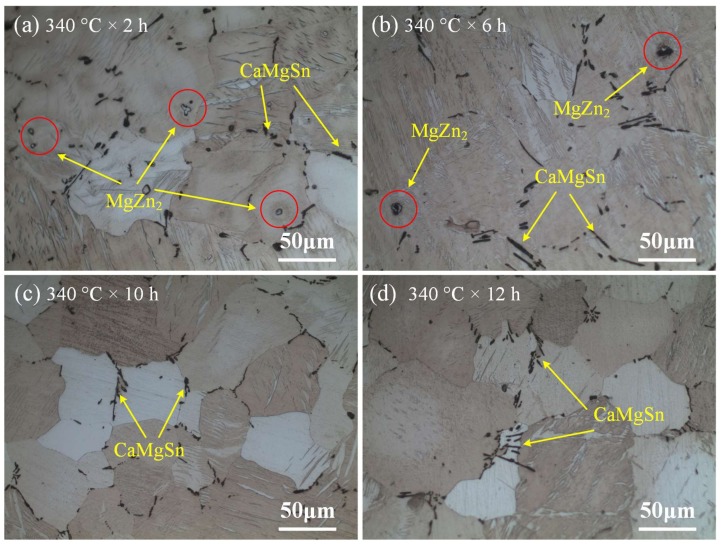
Optical microstructure of the samples for different homogenization time at the low-temperature stage of 340 °C in two-step homogenization.

**Figure 14:**
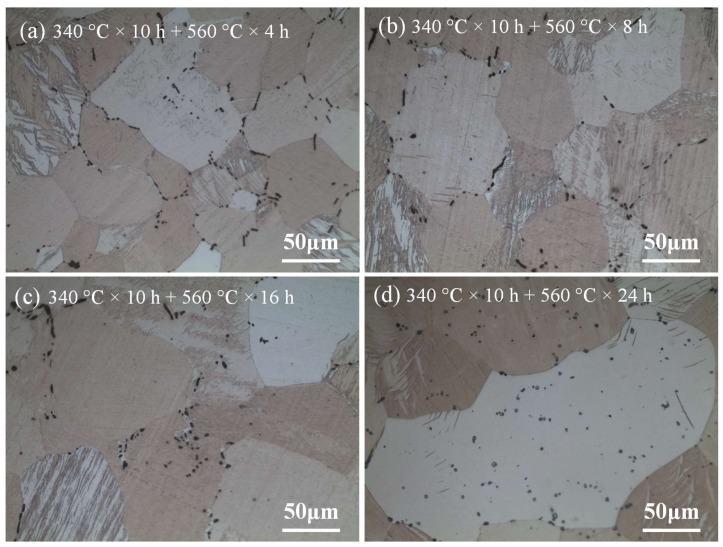
Optical microstructure of the samples for different homogenization time at the high-temperature stage of 560 °C in two-step homogenization.

At the low-temperature stage, the samples were heated to 340 °C and then kept for 2 h, 6 h, 10 h, and 12 h, respectively. Figure 13 shows the optical microstructure of the sample at low-temperature stage of 340 °C during two-step homogenization. Figure 13a,b show that the bright-white phase MgZn_2_ marked with the red circles decreases but still exists when the homogenization time increases from 2 h to 6 h, indicating incomplete dissolution of MgZn_2_ into the matrix. However, as shown in Figure 13c,d, with the further increase of the homogenization time, the bright-white MgZn_2_ disappears and only black CaMgSn can be observed, with grain size almost no change. Therefore, in two-step homogenization, the low-melting-point phase MgZn_2_ can be dissolved into the matrix for 10 h at the low-temperature stage of 340 °C. Figure 14 shows the optical microstructure of the sample at the high-temperature stage of 560 °C during two-step homogenization. As can be seen in Figure 14a–d, with the increase of homogenization time from 4 h to 24 h, the average grain size increases from about 73.6 μm to about 117.5 μm. Besides, the size and fraction of heat-resistant phase CaMgSn decreases and CaMgSn is gradually dissolved from long strip-like into dot-like. However, as shown in Figure 14c,d, when the homogenization time at the high-temperature stage exceeds 16 h, the pinning effect of heat-resistant phase CaMgSn is weakened sharply and the grain grows abnormally due to its dot-like morphology [38].

**Figure 15:**
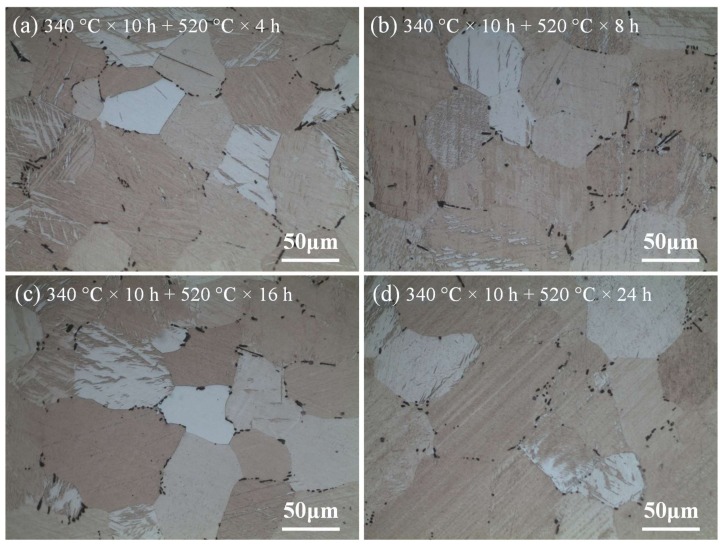
Optical microstructure of the samples for different homogenization time at the high-temperature stage of 520 °C in two-step homogenization.

Therefore, according to the above results, in order to achieve good homogenization effect without obvious grain growth at the same time, the temperature at the high-temperature stage is selected as 520 °C. Figure 15 shows the optical microstructure of the sample at the high-temperature stage of 520 °C during two-step homogenization. As can be seen in Figure 15a–c, with the increase of the homogenization time from 4 h to 16 h at high-temperature stage, the size and fraction of heat-resistant phase CaMgSn decrease gradually. Besides, the CaMgSn phase is gradually dissolved from long strip-like into short rod-like and dot-like without obvious grain growth due to the pinning effect of heat-resistant phase CaMgSn. However, the grains grow up more obviously when the homogenization time exceeds 16 h, as shown in Figure 15d. The average grain sizes of Figure 15a–d are about 71.3 μm, 72.1 μm, 73.5 μm and 77.2 μm, respectively. Thus, according to the morphology evolution of the heat-resistant phase CaMgSn and the difference of the grain size, the sample under the condition of two-step homogenization 340 °C × 10 h + 520 °C × 16 h achieves a good homogenization effect not only with uniform microstructure but also without obvious grain growth.

#### 3.2.2. Mechanical Properties and Fracture Behaviors

**Figure 16:**
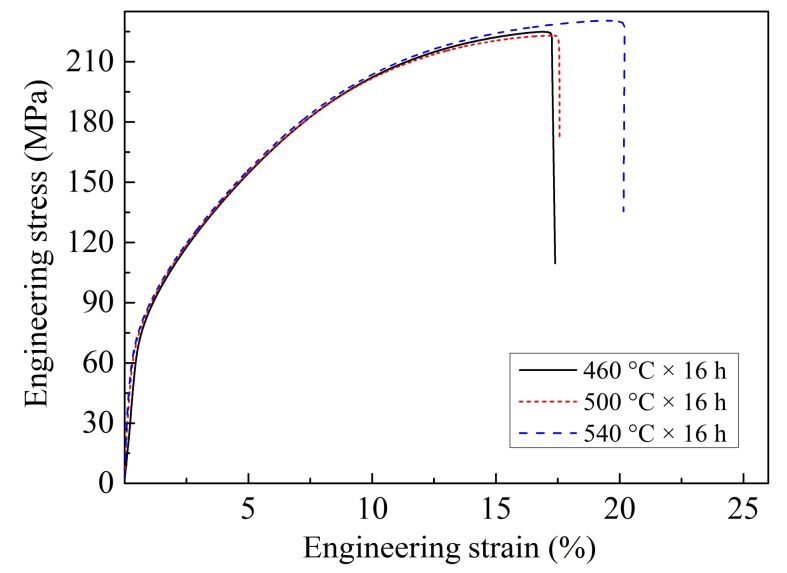
The engineering stress–strain curves of the as-homogenized ZTMX3100 alloy under different one-step homogenization conditions.

Figure 16 gives the tensile engineering stress–strain curves of the as-homogenized ZTMX3100 alloy under different one-step homogenization conditions and Table 4 gives the corresponding tensile properties. Compared with the as-cast alloy, the elongation of the as-homogenized alloy increases and the yield strength decreases. As can be seen from the one-step homogenization results shown in Figure 16, the elongation of the alloy gradually increases and the yield strength decreases with the increasing homogenization temperature. The sample homogenized at 540 °C presents a lower yield strength and higher elongation, with the YS, UTS, and EL of 71.0 MPa, 230.0 MPa, and 20.0%, respectively.

**Figure 17:**
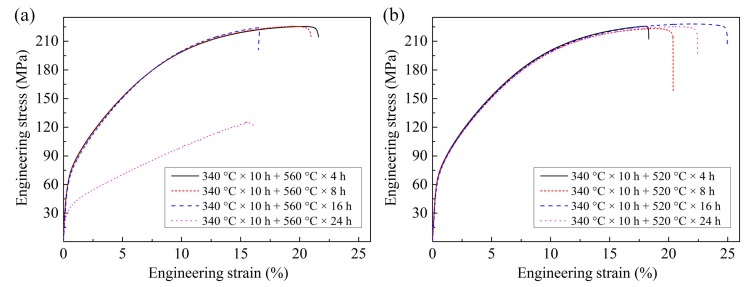
The engineering stress–strain curves of the as-homogenized ZTMX3100 alloy under different two-step homogenization conditions: (**a**) for high temperature stage of 560 °C; (**b**) for high temperature stage of 520 °C.

The results of two-step homogenization at 560 °C in Figure 17a indicate that the elongation of the alloy decreases with the increase of homogenization time. Especially, when the homogenization time is 16 h, the elongation of the alloy decreases obviously. In addition, both the strength and elongation of the alloy decrease sharply when the homogenization time is 24 h. The obvious degradation of mechanical properties is attributed to the abnormal grain growth, as shown in Figure 14c,d. According to Figure 17b and the corresponding tensile properties in Table 4, the elongation of the alloy firstly increases and then decreases, while the yield strength always decreases with the increasing homogenization time in two-step homogenization at 520 °C. Since the grain size increases with the increase of homogenization time, the yield strength of the alloy decreases gradually according to the Hall–Petch relation [39]. Besides, the size and fraction of heat-resistant phase CaMgSn decrease and the CaMgSn phase is gradually dissolved from long strip-like into short rod-like and dot-like without obvious grain growth, so the elongation increase gradually from 4 h to 16 h. However, when the homogenization time exceeds 16 h, the grains grow up obviously, which reduces the elongation of the alloy. Therefore, the sample homogenized under the condition of 340 °C × 10 h + 520 °C × 16 h achieves the optimum homogenization effect, with the YS, UTS, and EL of 67.0 MPa, 228.0 MPa, and 25.0%, respectively. Compared with the as-cast alloy, the yield strength of the alloy is reduced by 20.7% and the elongation is increased by 56.3%.

**Figure 18:**
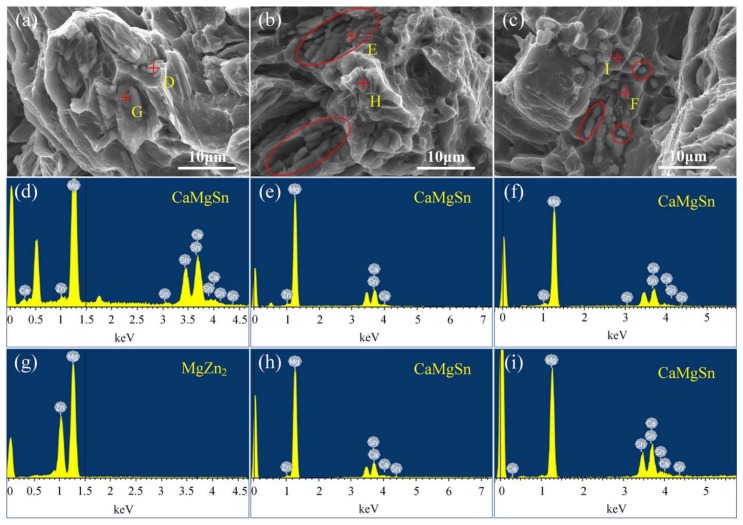
Magnified fracture SEM images and EDS results of the as-cast and as-homogenized alloys: (**a**,**d**,**g**) as-cast; (**b**,**e**,**h**) one-step homogenization (500 °C × 16 h); (**c**,**f**,**i**) two-step homogenization (340 °C × 10 h + 520 °C × 16 h).

Figure 18 shows the magnified fracture SEM images and EDS results of the as-cast and as-homogenized alloys, respectively. Figure 18a,d,g present the magnified fracture SEM images and EDS results of the as-cast alloy along the axial direction at 1/2 radius of the ingot. The magnified fracture SEM image in Figure 18a reveals that some micron-sized second phases are found in partial dimples of the tensile fractures. By combining with the EDS results in Figure 18d,g, MgZn_2_ and CaMgSn can be detected on the fracture surface. Figure 18b,e,h present the magnified fracture SEM images and EDS results of the alloy under the condition of one-step homogenization (500 °C × 16 h). After one-step homogenization treatment at 500 °C for 16 h, the coarse phase MgZn_2_ distributed along the grain boundary is dissolved into the matrix, and only CaMgSn can be detected on the fracture surface. Judging from the marked red-ellipses in Figure 18b, there are still some CaMgSn phases in the form of long strip-like after one-step homogenization at 500 °C for 16 h. Figure 18c,f,i present the magnified fracture SEM images and EDS results of the alloy under the condition of two-step homogenization (340 °C × 10 h + 560 °C × 16 h). According to the marked red ellipse and red circles in Figure 18b, only short rod-like and dot-like CaMgSn phase on the fracture surface can be detected, which is conducive to increase the elongation of the as-homogenized alloy. Therefore, the proper size and morphology of CaMgSn phase are conducive to increase the mechanical properties of the alloy.

In comparison with the as-cast alloy, the yield strength of the as-homogenized alloy decreases and its elongation increases. This is because the coarse second phase in the as-cast alloy leads to dislocation accumulation and stress concentration at the grain boundaries. The nucleation of microcracks firstly initiates on the interface between the second phase and the matrix, and the further gathering and grow-up of these microcracks promote the formation of cracks [33,40]. After the optimum two-step homogenization treatment, the coarse irregular phase MgZn_2_ is dissolved into the matrix and the heat-resistant phase CaMgSn is gradually dissolved from long strip-like into short rod-like and dot-like without obvious grain growth, so the crack-initiation is reduced. Therefore, the reasonable homogenization treatment can dissolve undesirable large second phases, eliminate the micro-segregation, and achieve a more uniform microstructure, which contributes to increasing the elongation of the as-homogenized alloy [41]. At the same time, homogenization treatment dissolves the low-melting-point phase MgZn_2_ and changes the morphology of heat-resistant phase CaMgSn, thus reducing the pinning effect of the second phase on dislocation motion and lowering the yield strength of the as-homogenized alloy [42,43]. As a result, the yield strength of the as-homogenized alloy decreases, but the elongation increases after homogenization treatment. Finally, for the ZTMX3100 alloy, the two-stage homogenization of 340 °C × 10 h + 520 °C × 16 h is determined as the optimum homogenization process. After the optimum homogenization treatment, the yield strength of the alloy is reduced by 20.7% and the elongation is increased by 56.3%, which is more favorable for the subsequent hot deformation processing.

## 4. Conclusions

In this paper, a new type of low-cost and RE-free Mg-3.36Zn-1.06Sn-0.33Mn-0.27Ca (wt %) alloy ingot with the diameter of 130 mm and the length of 4800 mm was fabricated by semicontinuous casting. The microstructure and mechanical properties of the ingot at different areas were systematically investigated. The microstructure and mechanical properties of the alloy under different homogenization conditions were studied. The following conclusions were drawn.

(1) The average grain size and the second phase size decrease, but the area fraction of the second phase increases gradually from the center to the surface of the ingot.
(2) The mechanical properties of the alloy in axial direction are slightly better than those in the radial direction, but independent on texture.
(3) The sample at 1/2 radius of the ingot along the axial direction exhibits the optimum comprehensive mechanical properties due to the combined effect of relatively small grain size, low second phase fraction, and uniform microstructure.
(4) In one-step homogenization, the morphology of CaMgSn and grain size has no obvious change when homogenized at 460 °C. While at 500 °C and 540 °C, the CaMgSn is dissolved gradually from long strip-like into short rod-like or chain-like with the increase of homogenization time, and the grains grow obviously when the homogenization time exceeds 16 h.
(5) In two-step homogenization, the low-melting-point phase MgZn_2_ can be dissolved into the matrix for 10 h at low-temperature stage of 340 °C. At high-temperature stage of 520 °C, with the increase of homogenization time, the elongation of the alloy firstly increases and then decreases, while the yield strength always decreases.
(6) The optimum homogenization process of ZTMX3100 alloy is 340 °C × 10 h + 520 °C × 16 h. After the optimum homogenization treatment, the proper size and morphology of CaMgSn phase are conducive to improve the microstructure uniformity and the mechanical properties of the alloy. Besides, the yield strength of the alloy is reduced by 20.7% and the elongation is increased by 56.3%.

## Figures and Tables

**Table 1:** Chemical compositions of the ingot analyzed at different areas.

Analyzed Element	Actual Composition (wt %)			
	Center	1/2 Radius	Near the Surface	Average
Zn	3.32	3.37	3.39	3.36
Sn	1.06	1.05	1.08	1.06
Mn	0.32	0.34	0.34	0.33
Ca	0.26	0.27	0.28	0.27
Mg	Bal.	Bal.	Bal.	Bal.

**Table 2:** Chemical composition of ingot in points marked as D, E, F and G in Figure 5c.

Point	Mg		Zn		Sn		Ca	
	wt %	at %	wt %	at %	wt %	at %	wt %	at %
D	64.35	85.76	2.99	1.48	25.47	6.95	7.18	5.81
E	76.94	91.45	4.05	1.79	14.56	3.54	4.46	3.21
F	43.94	67.82	56.06	32.18	-	-	-	-
G	99.13	99.68	0.87	0.32	-	-	-	-

**Table 3:** Tensile properties of the as-cast ZTMX3100 alloy along radial and axial directions at different areas.

Areas	YS (MPa)	UTS (MPa)	EL (%)
Along the radial direction			
Center	81.3 ± 2.0	210.0 ± 2.0	14.0 ± 0.4
1/2 radius	82.0 ± 1.1	207.0 ± 0.9	15.0 ± 0.7
Near the surface	75.0 ± 0.9	199.0 ± 1.3	13.5 ± 1.1
Along the axial direction			
Center	82.5 ± 0.5	212.0 ± 2.4	14.9 ± 0.9
1/2 radius	84.5 ± 1.2	210.0 ± 0.9	16.0 ± 0.8
Near the surface	76.0 ± 1.1	202.0 ± 1.5	14.2 ± 0.7

**Table 4:** Tensile properties of the as-homogenized ZTMX3100 alloy under different homogenization conditions.

Homogenization Conditions	YS (MPa)	UTS (MPa)	EL (%)
One-step homogenization			
460 °C × 16 h	78.0 ± 2.5	225.0 ± 0.5	17.0 ± 0.4
500 °C × 16 h	72.0 ± 0.8	223.0 ± 1.6	17.5 ± 0.8
540 °C × 16 h	71.0 ± 0.5	230.0 ± 1.0	20.0 ± 0.5
Two-step homogenization			
340 °C × 10 h + 560 °C × 4 h	69.0 ± 2.8	225.0 ± 0.7	21.5 ± 0.4
340 °C × 10 h + 560 °C × 8 h	67.0 ± 0.5	226.0 ± 1.2	21.0 ± 1.3
340 °C × 10 h + 560 °C × 16 h	68.0 ± 0.7	224.0 ± 0.8	16.3 ± 1.1
340 °C × 10 h + 560 °C × 24 h	32.0 ± 4.2	125.0 ± 3.8	15.3 ± 2.1
340 °C × 10 h + 520 °C × 4 h	72.0 ± 1.2	226.0 ± 0.9	18.5 ± 0.7
340 °C × 10 h + 520 °C × 8 h	70.0 ± 0.9	223.0 ± 1.5	20.5 ± 0.7
340 °C × 10 h + 520 °C × 16 h	67.0 ± 0.7	228.0 ± 0.7	25.0 ± 0.5
340 °C × 10 h + 520 °C × 24 h	66.0 ± 1.1	225.0 ± 1.4	22.5 ± 0.9
